# Mimicking Dolphins to Produce Ring Bubbles in Water

**DOI:** 10.3390/biomimetics1010006

**Published:** 2016-09-07

**Authors:** Philippe Lesage, Mohammed Kemiha, Souhila Poncin, Noël Midoux, Huai Z. Li

**Affiliations:** 1 Laboratoire Réactions et Génie des Procédés (CNRS, UMR 7274), Université de Lorraine, 1 rue Grandville, BP 20451, 54001 Nancy Cedex, France; souhila.poncin@univ-lorraine.fr; 2 Current address: Solvay, 39500 Tavaux Cedex, France; lesageph@gmail.com; 3 Current address: Centre for European Studies, Jagiellonian University, 31131 Krakow, Poland; mohammedkemiha@ces.uj.edu.pl

**Keywords:** ring bubble, generation mechanisms, dolphin, mimicking, hydrodynamics

## Abstract

Several studies report that dolphins, either captive or wild, can expel air from their blowhole to form ring bubbles. By means of an experimental setup consisting of an orifice coupled to a computer-controlled solenoid valve to simulate the dolphin’s blowhole and a vessel as the lungs, we examined the formation mechanism of a ring bubble under varying experimental conditions. With a better record than the most talented dolphin, we show that two aspects were demonstrated as essential to the successful generation of a ring bubble in water: the valve’s opening duration, and the pressure inside the vessel. The present findings suggest that during ring bubble production, dolphins are likely to anticipate their action by both adjusting a suitable air pressure inside their lungs and controlling their muscular flap for an adequate opening timing of their blowhole. This could provide some evidence in favour of suggested cetaceans’ self-control capacities.

## 1. Introduction

Air-breathing cetaceans always generate bubbles when expelling air in both solitary and social contexts. In particular, dolphins can release air from the blowhole, and the evacuated air rises to the free surface in a ring-shaped bubble [1,2,3,4]. The production of stable ring bubbles is a typical object manipulation behavior. There is some indication [5] in favour of a learning procedure to form ring bubbles. During ring bubble production, mothers attentively observe infants’ primary attempts at ring bubble generation and produce ring bubbles in their turn to provide an example for their infants. Thus, the quality of generated ring bubbles by infants is progressively improved, thanks both to this trial-and-error practice and the example from their mothers. This is a clear manifestation of cognitive capacities to some extent [6,7,8]. Reiss [9] proposed that, although expelling air in varied shapes through the blowhole, dolphins seem to have good control of both the shape and timing of the generation of ring bubbles. More recently, Ridgway et al. [8] similarly proposed that beluga whales (*Delphinapterus leucas*) mimicked human sounds by changing the nasal traction pressure and making suitable muscular adjustment of the vibrating phonic lips while over-inflating their vestibular sacs.

Previously, ring bubbles were observed by Walters and Davidson [10] in the course of their experimental investigation into the development of an initially spherical bubble of air in water. Other studies have only considered the rise of a ring bubble in the fluid dynamics literature [11,12]. The present work aims at understanding the fundamental mechanism of ring bubble generation and the corresponding levels of physical control mechanisms.

## 2. Materials and Methods

**Figure 1:**
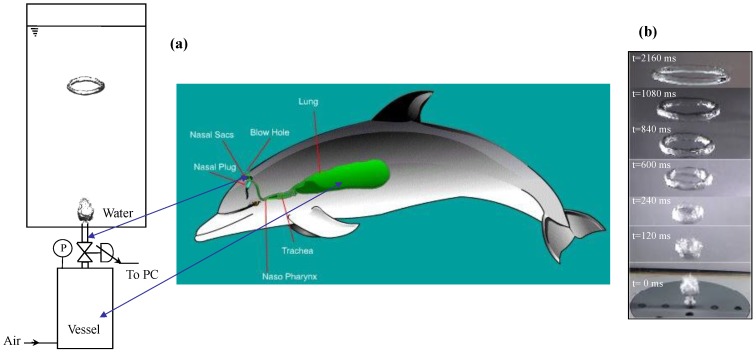
(**a**) Experimental setup to mimic the generation of ring bubbles by dolphins. (**b**) A complete sequence of the generation and rise of a stable ring bubble in water (relative pressure of 0.28 bar and opening duration of 0.030 s). The dolphin’s respiratory system reprinted [14]. P, pressure; PC, personal computer.

The respiratory system of a dolphin is mainly composed of the blowhole and lungs. Unlike other mammals who breathe through their nostrils and mouth, dolphins breathe through the blowhole that is centred at the top of their head. The dolphin must contract the muscular flap to open the blowhole that is naturally closed. In our mimicking experiments (Figure 1a), ring bubble formation was performed in a transparent Plexiglas tank of square cross section (0.20 m × 0.20 m) and 1.0 m high. The employed fluid was demineralized water at constant temperature (293 K). A 1 × 10^−3^ m^3^ pressurized vessel was used to simulate the dolphin’s lungs. A pressure transducer (Lucas, TX, USA) with an accuracy of 50 Pa indicated the air pressure inside the vessel. To mimic the blowhole, air bubble generation was done through a submerged cylinder made of a polyvinyl chloride (PVC) pipe with an inner diameter of 2 mm, a wall thickness of 2 mm, and a length of 50 mm, at the bottom section. The orifice had a flat exit. A computer-controlled micro-solenoid valve of high precision (Sirai, Bussero, Italy), mounted between the vessel and the orifice to mimic the dolphin’s muscular action, permitted bubbles to be injected with a desired opening duration [13]. Different opening duration and vessel pressures produced different bubble volumes. Cylinders with other inner diameters were also tested: 1 mm gave similar results as 2 mm, with smaller ring bubbles; however, the gravitational backflow of water in greater diameters tended to produce stable ring bubbles.

A high-speed video camera Phantom v711 ranging from 20 to 10,000 frames/s (Visions Research, Del Mar, CA, USA) was used to visualize the formation of a ring bubble at the orifice. Flow velocity fields around a ring bubble in liquid were quantified using particle image velocimetry (PIV, Dantec Dynamics, Denmark). Laser illumination sheets were generated by means of two pulsed neodymium-doped yttrium aluminium garnet (Nd:YAG) lasers (Dantec Dynamics, Skovlunde, Denmark) arranged side-by-side and crossing the vertical axis of the ring bubble. Unlike the high-speed camera, the PIV device had a limited measure rate of 25 frames per second. Fluorescent Rhodamine B beads of 5 µm (Dantec Dynamics) were added to the liquid as seeding particles. An orange filter placed in front of the camera avoided the reflection of the lasers on the bubbles and allowed only the passage of fluorescent light of the particles. The camera, placed orthogonal to the laser sheet, took two successive images, each at the intensity peak of the laser impulse intensity. These images were divided into several thousand small interrogation areas of 32 × 32 pixels in size. A cross-correlation was then carried out on the two corresponding interrogation areas. This correlation then computed both the liquid flow field and the rise velocity of the ring bubble.

The typical, complete sequence of the generation of a stable ring bubble in water—from the formation of a ring bubble near the injection orifice to its progressive rise towards the free surface—is shown in Figure 1b and Video S1. Not having the same scale as the dolphin’s respiratory system, our experimental setup aims mainly at mimicking the generation mechanism of ring bubbles.

## 3. Results

**Figure 2:**
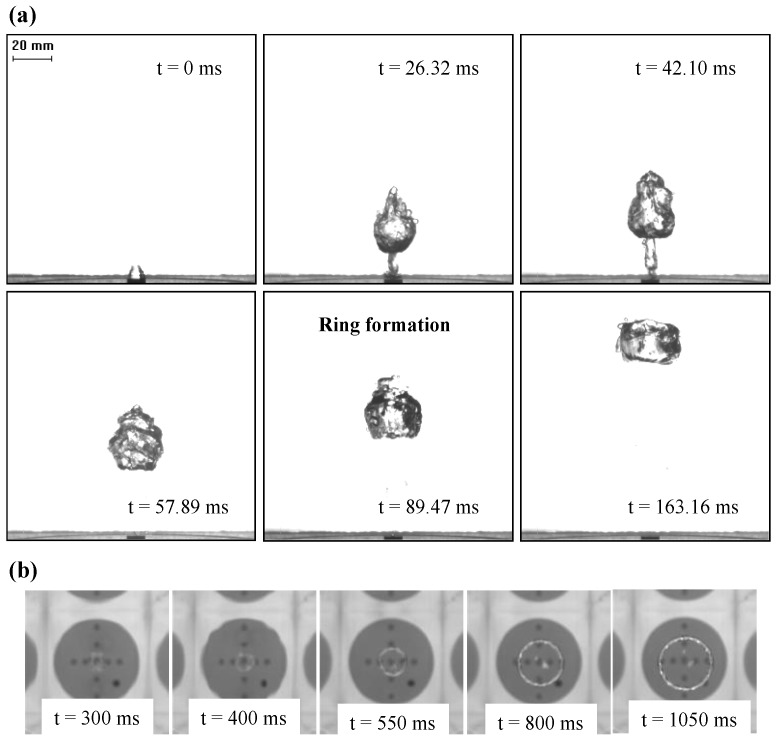
(**a**) Front view: temporary sequence of the successful formation of a ring bubble recorded with high-speed camera at 950 frames/s with relative pressure of 0.28 bar and opening duration of 0.030 s during its initial stage (cf. time scale indicated) before reaching a stable ring; (**b**) Top view of the ring bubble during its rise after having reached its stable ring shape.

**Figure 3:**
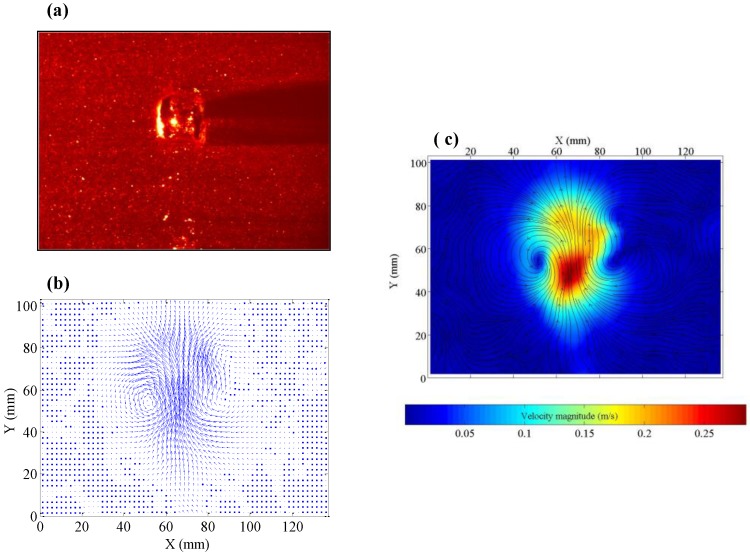
Complex flow around a ring bubble at its critical forming moment by means of the particle image velocimetry (PIV) device (relative pressure of 0.55 bar and opening duration of 0.015 s). (**a**) Brut image with the bubble in water in the presence of seeding fluorescent particles and a laser beam coming from the left. The critical moment of ring formation with the penetration of a liquid tongue at the top of the bubble can be observed. Both (**b**) the flow field and (**c**) streamlines identify the central, however dissymmetrical liquid jet contour and edge recirculation as the main factors affecting the generation of a ring structure.

The generation mechanism of a ring bubble is clearly shown by the high-speed camera recordings with a front view (Figure 2a), during its transient formation regime and a top view (Figure 2b), for the rise period after reaching a stable ring shape. In particular, the key moment in ring bubble formation is the rupture of the central air core due to a liquid jet as illustrated by the PIV flow measurement (Figure 3). In fact, on detaching from the orifice, the bubble moves with high inertia, but after passing through a short distance, the bubble front slows down due to the viscous drag of the water. The liquid inertia due to the sudden injection of a gas bubble can form a central tongue that pushes into the bubble from below. When the bottom interface impinges on the upper one, the bubble’s morphology changes to form a ring, caused by the penetration of the liquid tongue. In addition, the vortex sheet which expands at the edges has the same sense of circular motion as the central liquid tongue to facilitate the ring formation.

**Figure 4:**
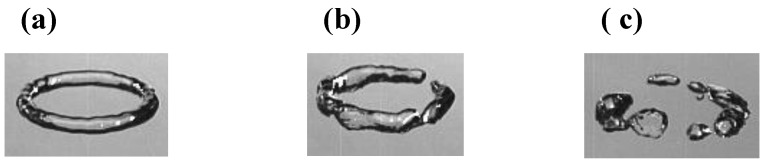
Three-point scale to assess the quality of ring bubbles generated by our mimicking setup shown in Figure 1a: (**a**) Successful; (**b**) Partial; (**c**) Poor.

McCowan et al. [5] proposed a four-point scale to assess the quality of the ring bubbles immediately after production during their observation with four juvenile dolphins: poor for broken ring; fair for partial ring; good as a complete but wide ring; and finally excellent for complete and tiny ring. In our study, a complete and wide ring bubble can always become tiny during the rise, as also reported in the literature [11,12]. Thus, we propose the use of a three-point scale to assess the quality of ring bubbles by grouping the good and excellent categories (i.e., successful, partial and poor) as shown in Figure 4.

The experimental data and the quantitative observation of four juvenile dolphins [5] are collected in Table 1 and Table 2, respectively. At total, we repeated 400 experiments with a time interval of 5 min to allow the stabilisation of the water flow after the passage of a bubble. As Supplementary Materials, high-speed video recordings are provided to illustrate the formation of both successful and poor ring bubbles (Videos S2 and S3, respectively).

These data show that by means of a well-controlled mimicking setup, we can certainly surpass the most talented dolphin in the performance of ring bubble production with the highest successful rate of 52%. However, the production of ring bubbles is not always successful, mainly due to the dissymmetric flow turbulence and high instability at the bubble interface, as shown in Figure 2 and Figure 3. Under the higher pressure inside the vessel, the increase of the opening duration intensifies the flow turbulence to make the ring structure unstable. On the other hand, the impulse momentum quantity of the liquid tongue is not sufficient to correctly penetrate the air core centre to form a regular ring shape if the pressure is too small and the opening duration too short. A suitable compromise appears to be required between the injected bubble volume and the impulse momentum quantity. Our experimental investigations suggest that the optimal range for both the relative pressure inside the vessel and the opening duration is 0.28 to 0.55 bar and 0.015 to 0.030 s, respectively, to guarantee a greater success rate. The physical mechanism of the formation of a bubble ring described here has some similarities to the expanding rings surrounding the mushroom-shaped cloud created by an above-ground nuclear detonation, or by firing certain types of artillery through a fast opening based on the sudden turbulence injection [11,12].

Unlike a gas–gas system such as smoke rings, which are usually formed when a puff of smoke is suddenly injected into clear air, the turbulence induced by the air in the ring bubble formation in water as gas-liquid system should be more intensive to overcome the water drag that is much higher than that opposed by the still air surrounding the outer parts of the puff for smoke rings. Due to the significant difference of both density and viscosity between air and water, an excessive turbulence during the initial stage of ring bubble generation (as shown in Figure 2), might explain why it is difficult to achieve a systematically reliable success rate for either dolphins or our mimicking setup.

## 4. Discussion

As shown in Figure 2 and Figure 3, as well as in the video recording included in the Supplementary Materials, the flow turbulence and bubble’s interfacial instability render beyond the reach of modeling and computation for a quantitative description. The measurements of the flow fields only in the water phase (as shown in Figure 3) did not allow the formation of successful or unsuccessful ring bubbles to be distinguished, as this is the very initial stage with excessive turbulence. Ideally, the flow fields should be measured in the gas phase too but it is still impossible at present. Detailed descriptive data are also missing in the literature with regard to the ring bubbles produced by dolphins. However, it is clearly demonstrated that the successful generation of a stable ring bubble stems from the dual action of suitable pressure inside the vessel (dolphin’s lungs) and the well-controlled opening duration of the electronic valve (dolphin’s muscular action). Thus, dolphins do not blow ring bubbles at random. Our experimental investigation suggests that the generation of stable ring bubbles may need certain experience and forethought not only by juvenile but also older dolphins as reported in the literature [4]. The present results points towards the assumption that dolphins monitor the quality of generated ring bubbles and exert anticipatory planning of certain level prior to ring bubble generation. The existing literature [6,7] suggests that dolphins do have a number of cognitive abilities, which might be consistent with our hypothesis. Certainly, the dolphins’ blowhole is larger than the PVC pipe (with an inner diameter of 2 mm) used in this work. The main objective of the present study was to investigate the mechanism of ring bubble generation. Thus, the scaling plays a secondary role as shown by the similarity between ring bubbles in water, mushroom clouds, and smoke rings. In particular, a rigid pipe with a larger inner diameter would induce some instabilities, as the capillary forces can no longer prevent water from entering the pipe.

**Figure 5:**
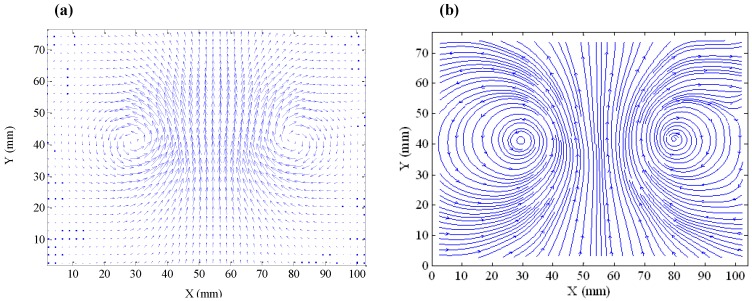
(**a**) Flow field and (**b**) streamlines around a stable rising ring bubble at 0.45 m above the orifice with relative pressure of 0.28 bar and opening duration of 0.030 s.

During the rise of a stable ring bubble, the plane of the ring is usually perpendicular to the direction of the rising. When the ring bubble moves progressively upwards, the rise velocity diminishes with the increase of the ring diameter and the shrinkage of the cross-section of the air core. The perfect symmetric flow field (Figure 5a) around a stable ring bubble measured by the PIV device reveals the fluid recirculation structure around the air core (Figure 5b).

## 5. Conclusions

Our study provides some insight into the level of control that dolphins may exercise on themselves as well as on their surrounding water medium. Clearly, the complex mechanisms behind some of the apparently trivial behaviors of cetaceans could be understood better still by mimicking them under well-controlled operating conditions. Although our experimental device needs consideration of alternatives and improvements, the optimal conditions for ring bubble generation (as shown in Table 1)—pressure ranging from 0.28 to 0.55 bar and time intervals from 0.015 to 0.030 s in our work—correspond quite well to previously reported pressure (0.27 to 0.66 bar) and intervals between bursts (0.05 to 0.5 s) with fundamental frequencies of 200 to 300 Hz for human speech mimicry by a beluga whale [8]. It is worth noting that all that is known is that air is ejected from the dolphins’ blowhole. Whether the lungs or bursa are the source of the air is still a matter of conjecture without more data on the animal. Further investigation would be required for the analysis of the anatomy of a dolphin with respect to the ring bubble formation.

In addition to the biomimetic aspect as mentioned above, the successful production of ring bubbles in water or other liquids could bring new challenges for modeling the rising morphological evolution of classical bubble shapes [15,16]. The peculiar ring bubble structure, along with the eddy vortex, would be useful to intensify the gas–liquid mass transfer [17] encountered in numerous industrial applications, such as harmful gas removal and CO_2_ capture.

## Figures and Tables

**Table 1:** Quality of ring bubble production according to the three-point scale (Figure 4) with our mimicking setup operating under different conditions.

Relative Pressure (bar)	Opening Duration (s)	Bubble Volume (×10^9^ m^3^)	Success (%)	Partial (%)	Poor (%)
0.14	0.015	1.90	29.0	42.2	28.8
0.14	0.030	3.42	32.0	19.5	48.5
0.14	0.040	4.75	29.0	30.0	41.0
0.28	0.015	6.08	34.0	26.5	39.5
0.28	0.030	10.26	52.0	24.5	23.5
0.28	0.040	12.35	35.5	23.0	41.5
0.55	0.015	10.17	50.0	34.0	16.0
0.55	0.030	17.10	43.0	29.0	28.0
0.55	0.040	20.05	29.5	39.0	31.5

**Table 2:** Existing data of ring bubble production by juvenile dolphins in the literature [5].

Juvenile Dolphin	Tentative Number	Success (%)
Avalon	88	31.8 *
Brisbee	13	30.8
Liberty	20	33.3 *
Norman	30	6.7 *

* Some data were missing during observations [5].

## Supplementary Materials

The following are available online at http://www.mdpi.com/2313-7673/1/1/6/s1, Video S1: Formation Rise—A complete sequence of the generation and rise of a ring bubble in the tank recorded with an average speed camera (relative pressure of 0.28 bar and opening duration of 0.030 s), Video S2: Successful Ring—The formation in slow motion of a perfect ring bubble of successful quality recorded with a high-speed camera at 950 frames/s (relative pressure of 0.55 bar and opening duration of 0.015 s), Video S3: Poor Ring—The formation in slow motion of a broken ring bubble of poor quality recorded with a high-speed camera at 950 frames/s (relative pressure of 0.55 bar and opening duration of 0.015 s).
