# Supplementary figures and images for: Mimicking Dolphins to Produce Ring Bubbles in Water
Source: Biomimetics (Basel). 2016 Sep 7;1(1):6. doi: 10.3390/biomimetics1010006 (PMC6477604; doi:10.3390/biomimetics1010006)

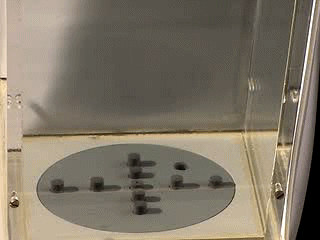

Supplement: Supplementary file 1 [file biomimetics-01-00006-s001.zip › biomimetics-01-00006-supplementary/VideoS1_FormationRise.gif]

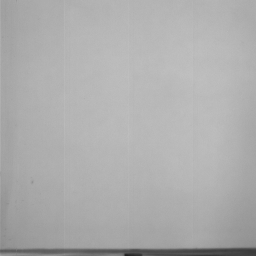

Supplement: Supplementary file 1 [file biomimetics-01-00006-s001.zip › biomimetics-01-00006-supplementary/VideoS2_SuccessfulRing.gif]

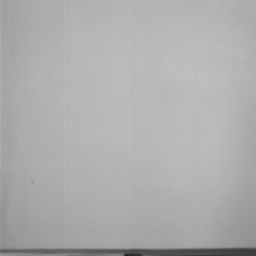

Supplement: Supplementary file 1 [file biomimetics-01-00006-s001.zip › biomimetics-01-00006-supplementary/VideoS3_PoorRing.gif]
